# Antibacterial and Hemolytic Activity of *Crotalus triseriatus* and *Crotalus ravus* Venom

**DOI:** 10.3390/ani10020281

**Published:** 2020-02-11

**Authors:** Adrian Zaragoza-Bastida, Saudy Consepcion Flores-Aguilar, Liliana Mireya Aguilar-Castro, Ana Lizet Morales-Ubaldo, Benjamín Valladares-Carranza, Lenin Rangel-López, Agustín Olmedo-Juárez, Carla E. Rosenfeld-Miranda, Nallely Rivero-Pérez

**Affiliations:** 1 Universidad Autónoma del Estado de Hidalgo, Área Académica de Medicina Veterinaria y Zootecnia, Instituto de Ciencias Agropecuarias, Rancho Universitario Av. Universidad km 1, EX-Hda de Aquetzalpa, Tulancingo, Hidalgo 43600, Mexico; adrian_zaragoza@uaeh.edu.mx (A.Z.-B.); ubaldolizet8@gmail.com (A.L.M.-U.); ralolenin@gmail.com (L.R.-L.); 2 Universidad Autónoma del Estado de Hidalgo, Área Académica de Biología, Instituto de Ciencias Básicas e Ingeniería. Carretera Pachuca-Tulancingo S/N Int. 22 Colonia Carboneras, Mineral de la Reforma, Hidalgo 42180, Mexico; saudy.fa@gmail.com (S.C.F.-A.); profe_3192@uaeh.edu.mx (L.M.A.-C.); 3 Facultad de Medicina Veterinaria y Zootecnia Universidad Autónoma del Estado de México, El Cerrillo Piedras Blancas, Toluca 50295, Mexico; benvac2004@yahoo.com.mx; 4 Centro Nacional de Investigación Disciplinaria en Salud Animal e Inocuidad (CENID SAI-INIFAP), Carretera Federal Cuernavaca-Cuautla No. 8534 / Col. Progreso, Jiutepec 62550, Morelos, Mexico; aolmedoj@gmail.com; 5 Facultad de Ciencias Veterinarias, Universidad Austral de Chile, Isla Teja s/n, Casilla 567, Valdivia, Chile; crosenfe@uach.cl

**Keywords:** *Crotalus ravus*, Crotalus triseriatus, venom, antibacterial activity, *Pseudomonas aeruginosa*, hemolytic activity

## Abstract

**Simple Summary:**

Rattlesnakes (*Crotalus ravus* and *Crotalus triseriatus*) have some compounds that resemble polypeptides and proteins in their venoms which can be used in therapeutic treatment as antibacterial compounds. The aim of the present study is to evaluate the antibacterial and hemolytic activity of two rattlesnake venoms. The results of the present study indicate that the evaluated venoms have bactericidal activity against *Pseudomonas aeruginosa*, an important bacterium that affects animals and humans, thereby providing a new and efficient treatment alternative against this pathogenic bacterium.

**Abstract:**

Rattlesnakes have venoms with a complex toxin mixture comprised of polypeptides and proteins. Previous studies have shown that some of these polypeptides are of high value for the development of new medical treatments. The aim of the present study is to evaluate, in vitro, the antibacterial and hemolytic activity of *Crotalus triseriatus* and *Crotalus ravus* venoms. A direct field search was conducted to obtain *Crotalus triseriatus* and *Crotalus ravus* venom samples. These were evaluated to determine their antibacterial activity against *Escherichia coli*, *Staphylococcus aureus* and *Pseudomonas aeruginosa* through the techniques of Minimum Inhibitory Concentration (MIC) and Minimum Bactericidal Concentration (MBC). Hemolytic activity was also determined. Antibacterial activity was determined for treatments (*Crotalus triseriatus* 2) CT2 and (*Crotalus ravus* 3) CR3, obtaining a Minimum Inhibitory Concentration of 50 µg/mL and a Minimum Bactericidal Concentration of 100 µg/mL against *Pseudomonas aeruginosa*. CT1 (*Crotalus triseriatus* 1), CT2, and CR3 presented hemolytic activity; on the other hand, *Crotalus ravus* 4 (CR4) did not show hemolytic activity. The results of the present study indicate for the first time that *Crotalus triseriatus* and *Crotalus ravus* venoms contain some bioactive compounds with bactericidal activity against *Pseudomonas aeruginosa* which could be used as alternative treatment in diseases caused by this pathogenic bacterium.

## 1. Introduction

Rattlesnakes are a species widely distributed through Mexico, occupying practically the whole territory. There exists a great variety of these species, among them, are *Crotalus triseriatus,* distributed in the States of Veracruz, Puebla, Tlaxcala, México, Morelos, and Michoacán and *Crotalus ravus,* which occupies the States of Morelos, México, Puebla, Tlaxcala, Guerrero, Oaxaca, and Hidalgo. These species are primarily recognized for their characteristic hemotoxic venoms [1,2,3].

Crotalid venoms are comprised mainly of enzymes that cause severe local inflammation, necrosis, hemorrhagic syndromes, and neurological manifestations. These responses would typically help rapid prey subjugation or capture, as well as serve as a defense mechanism [4]. 

Animal venoms, including that of snakes, are complex mixtures of bioactive compounds that contain large amounts of proteins, peptides, and small molecules that can be considered for use in a wide range of medical applications [5,6].

There are several examples in the development of treatments derived from snake venom compounds. One of the most widely known is Capoten^®^, a hypotensive agent, used for the treatment of congestive heart failure, diabetic nephropathy, and heart attacks. Another known example is Viprinex^®^, developed to treat acute strokes [7,8].

Aside from their qualities as potential therapeutic agents, venoms are currently considered as possible sources of molecules with antibacterial activity [9]. This, in fact, has a great impact on public health especially due to the increase of antibacterial resistant bacteria. 

In 2017 the World Health Organization (WHO) compiled a list of antibiotic-resistant priority pathogens, among which, were the Gram-negative bacteria *Escherichia coli* and *Pseudomonas aeruginosa* bacteria resistant to carbapenems, and *Staphylococcus aureus* resistant to methicillin and vancomycin [10]. Due to the increased antibiotic resistance found in these pathogens, the aim of the present study was to evaluate, in vitro, the antibacterial and hemolytic response of *Crotalus triseriatus* and *Crotalus ravus* venoms on bacteria of public health importance.

## 2. Material and Methods

### 2.1. Field Sampling

Two field outings were carried out per month during each of the months of August, September, October and November 2018 in the state of Hidalgo, Mexico; covering the municipalities of Acatlán, Almoloya, Cuautepec de Hinojosa, Mineral del Chico, Mineral del Monte, Santiago Tulantepec, Singuilucan, Tula de Allende and Zacualtipán.

A direct search was conducted according to the methodology described by McDiarmid et al. in 2012. The rattlesnakes were trapped in accordance with the official norms for wildlife protection (NOM-059-SEMARNAT-2010) established by the government of Mexico and with a scientific collecting permit issued by General Directorate of Wildlife of the Secretariat of Environment and Natural Resources of Mexico (Office N° SGPA/SGVS/003613/18) [11,12]. 

### 2.2. Obtaining Venom Samples

Four samples were collected in the field, two of them belonging to the species *Crotalus triseriatus* (CT1 and CT2) and the remaining from the species *Crotalus ravus* (CR3 and CR4). A record of each individual was noted.

Once the samples were obtained, they were subjected to a lyophilization process and kept at −70 °C until further evaluation.

### 2.3. Antibacterial Activity

The venom’s antibacterial activity was determined through the Minimum Inhibitory Concentration (MIC) and the Minimum Bactericidal Concentration (MBC) procedures, in accordance with the CLSI guidelines and with the standards published by Olmedo-Juárez et al., in 2019 and by Morales-Ubaldo et al., in 2020 [13,14,15].

*Escherichia coli* ATCC^35218^, *Pseudomonas aeruginosa* ATCC^9027^, and *Staphylococcus aureus* ATCC^6538^ strains were used to perform the evaluation. These samples were the same which were reactivated from cryopreservation in Müller–Hinton agar (BD Bioxon, Heidelberg, Germany) through simple strain technique to obtain isolated colonies. A Gram staining was performed to corroborate their morphology. 

Once the purity was confirmed, one colony of each strain was inoculated in nutritive broth (BD Bioxon), and incubated under constant agitation at 70 rpm for 24 h at 37 °C. The bacterial cell suspension was adjusted to a 0.5 McFarland (Remel, R20421, Kansas, U.S.A.) standard (approximately 1.5 × 10^6^ Colony Forming Units (CFU) per mL).

#### 2.3.1. Minimal Inhibitory Concentration (MIC)

Micro-dilution was used to determine the MIC, evaluating different venom concentrations (100, 50, 25, 12.5, 6.25, 3.12, 1.56, 0.78 µg/mL). 

In a sterile 96- well plate, 100 µL of each venom concentrations were added along with 10 µL of bacterial cell suspension previously adjusted to a 0.5 McFarland standard. The plates were incubated at 37 °C for 24 h at 70 rpm. Kanamycin (AppliChem 4K10421, Darmstadt, Germany) was used as a positive control (128 to 1 µg/mL) and nutritive broth as the negative control. Treatments were evaluated by triplicate.

After incubation 20 μL of a 0.04% (*w*/*v*) p-iodonitrotetrazolium (Sigma-Aldrich I8377, Missouri, U.S.A.) solution was added into each well and incubated for 30 min. The MIC was determined by the concentration at which the solution turned to a pinkish color.

#### 2.3.2. Minimal Bactericidal Concentration (MBC)

After incubation and previous addition of p-iodonitrotetrazolium, 5 μL from each well was inoculated in Müller–Hinton agar (BD Bioxon) and incubated at 37 °C for 24 h. The MBC was considered as the lowest concentration where no visible growth of the bacteria was observed on the plates.

### 2.4. Indirect Hemolytic Activity 

In accordance with the protocols described by Pirela et al., in 2006 with modifications, the venom’s indirect hemolytic activity was evaluated [16]. A donor donkey blood sample was collected. The blood sample was stored in 10 mL sodium citrate (3.2%) tubes (DB Vacutainer) and in 3 mL EDTA (10.8 mg) tubes (BD Vacutainer).

Blood agar was used (Merck©, Darmstadt, Germany). To obtain plates with 8% blood concentration, 250 mL of agar base was prepared, and 20 mL of blood was added. 

One hundred micrograms (100 µg) of each treatment were weighed out (lyophilized venom) and reconstituted in 1 mL of nutritive broth (BD Bioxon). Dilutions were made (100, 50, 25, 12.5, 6.25, 3.12 µg/mL) from this concentrated solution for further evaluation.

Four wells were made (6 mm diameter) on the plate’s surface. Twenty micrograms (20 µL) were added of each concentrate to be evaluated. Treatments were performed by triplicate. Tween 80 at 100% (Sigma-Aldrich) and nutritive broth (BD Bioxon) were used as positive and negative controls, respectively. Plates were incubated for 24 h at 37 °C. Once the incubation period elapsed, hemolysis halos were measured (mm).

### 2.5. Statistical Analysis.

Obtained data were analyzed using two-way variance analysis (ANOVA) and a means comparison by Tukey at a significance level of 0.05% through Minitab 18 statistical package [17].

## 3. Results and Discussion

### 3.1. Individuals Data

**Figure 1:**
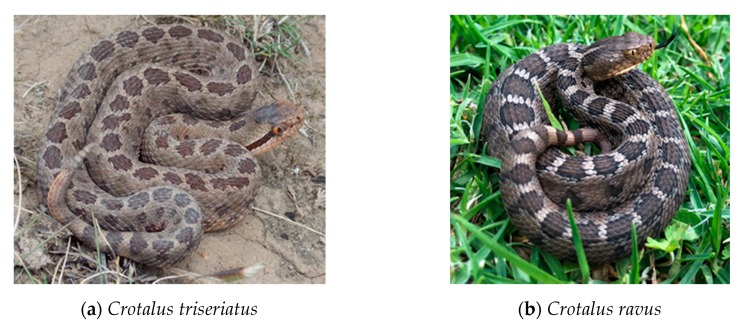
Captured species (**a**) *Crotalus triseriatus* and (**b**) *Crotalus ravus.*

A record of each individual was made with the following information: length, weight, age, and gender (Table 1). The characteristics of the rattlesnakes in the study coincided with those reported by Campbell and Lamar in 2004 [1], as seen in Figure 1.

### 3.2. Antibacterial Activity

**Figure 2:**
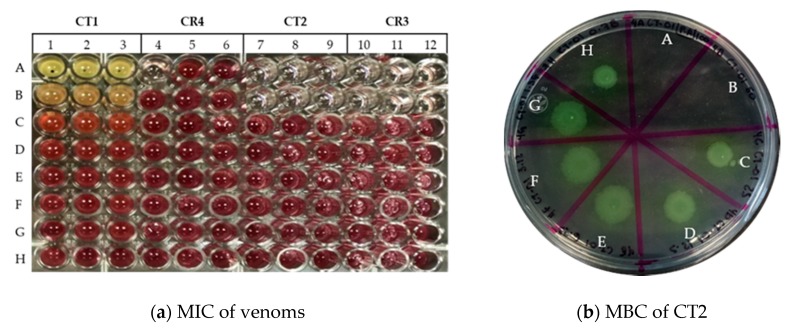
Antibacterial activity of rattlesnake’s venoms against *P. aeruginosa*: (**a**) columns 1–3, CT1 from 100 at 0.78 µg/mL, columns 4–6, CR4 from 100 at 0.78 µg/mL, columns 7–9, CT2 from 100 at 0.78 µg/mL, columns 10–12, CR3 from 100 at 0.78 µg/mL. The MIC value is read at the minimal concentration in which the color changes to pink; (**b**) Plate with *P. aeruginosa* + CT2 in Müller–Hinton agar; **A** CT2 to 100 µg/mL, **B** CT2 to 50 µg/mL, **C** CT2 to 25 µg/mL, **D** CT2 to 12.5 µg/mL, **E** CT2 to 6.25 µg/mL, **F** CT2 to 12.5 µg/mL, **G** CT2 to 6.25 µg/mL, **H** CT2 to 0.78 µg/mL. The MBC is read to the lowest concentration where no visible growth of the bacteria.

A MIC of 50 µg/mL and an MBC of 100 µg /mL were determined as effective for treatments CT2 and CR3 over *P. aeruginosa* (Table 2, Figure 2). Nevertheless, antibacterial activity was not detected for *E. coli and S. aureus*.

It was determined that the antibacterial response seen in treatments CT2 and CR3 were bactericidal, since the relation between MIC and MBC is less than 4, in accordance with González-Alamilla et al., in 2019 [18].

Boda et al., in 2019 evaluated the antibacterial activity of eleven crude venoms from different snake species including *Crotalus atrox* and *Crotalus polystictus* against *Staphylococcus aureus, Escherichia coli* and *Pseudomonas aeruginosa* among others, at varied concentrations of 500 to 1.95 µg/mL, determining a MIC and MBC of 125 and 500 µg/mL against *S. aureus* for *Crotalus atrox* and *Crotalus polystictus*, respectively. In the present study, antibacterial activity was not found for *S. aureus* and *E. coli* but was determined for *Pseudomonas aeruginosa,* obtaining a MIC of 50 and a MBC of 100 µg/mL for *C. triseriatus* and *C. ravus* (CT2 and CR3). According to Boda et al., 2019, the antibacterial activity of venoms from viperid species is probably due to their content of proteins with proteolytic activity [19].

Samy et al., in 2014, evaluated CaTx-II a toxin isolated from *Crotalus adamanteus* venom, determining a MIC of 7.8 µg/mL for *S. aureus* and 62.5 to 125 µg/mL for *P. aeruginosa*. Oguiura et al., in 2011, evaluated crotamine, a myotoxin from *Crotalus durissus* venom against different bacteria strains which included *E. coli*, *S. aureus*, and *P. aeruginosa*. They report a MIC of 100 µg/mL for *E. coli* and >200 µg/mL for the other two [20,21], a contrast with the results obtained in our present study since the antibacterial activity was not determined for *E. coli* or *S. aureus*. Since it was determined that a MIC of 50 µg/mL from *C. triseriatus* and *C. ravus* (CT2 and CR3) occurred in crude venom, the activity could be attributed to the presence of these bioactive compounds in the venom of the individuals used for this evaluation since both compounds were isolated from snake venom of the same genus (*Crotalus*). 

Although the aim of the study did not include identifying the venom’s active mechanism, it has been reported that phospholipase A_2_ (CaTx-II) interacts with lipopolysaccharide (LPS), particularly with lipid A, a Gram-negative bacteria component, causing membrane permeabilization. Crotamine also has effects over some bacteria through membrane permeabilization, so it could be suggested that CT2 and CR3 treatments antibacterial activity is related to this mechanism [21,22].

In this respect, the efficiency of these compounds, specially phospholipase A_2_ against antibiotic-resistant bacteria, holds promise for biotechnological applications, in this case, new medical treatment alternatives, however, it should be understood there are different antibacterial activity mechanisms from venom-based drugs [23,24].

In accordance with WHO, *P. aeruginosa* actually is in the critical priority group of the list of antibiotic-resistant pathogens. WHO has been expressing its interest by promoting the research and development of new antibiotics for this bacterium [10]. These results obtained herein show that CT2 and CR3 treatments demonstrated bactericidal activity against this pathogen showing its importance, since rattlesnake venoms or compounds thereof could be used to develop effective therapeutic agents to treat infections caused by *P. aeruginosa*. 

### 3.3. Hemolytic Activity

**Figure 3:**
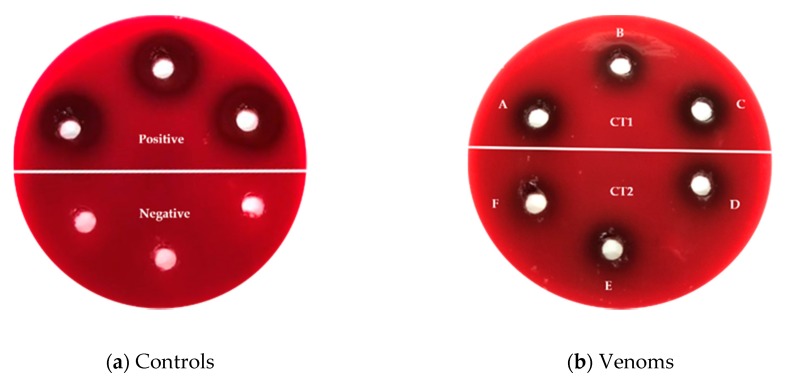
Indirect hemolytic activity of the rattlesnake’s venoms: (**a**) hemolytic activity of controls, positive Tween 80, negative nutritive broth; (**b**) hemolytic activity of venoms **A**, **B**, **C** 100 µg/mL of CT1; **D**, **E**, **F** 100 µg/mL of CT2.

With respect to the hemolysis produced, the generated halos showed significant statistical differences between them (*p* < 0.05) (Table 3). It was observed that CT1, CT2, and CR3 showed the highest hemolytic potential and there were no statistically significant differences between them at 100 µg/mL concentration compared with the other treatments (Figure 3).

Macías-Rodríguez et al., in 2014 [25] evaluated the hemolytic activity of *Crotalus molossus* venom (*C. molossus molossus* and *C. molossus nigrescens*) at a 50 µg/mL concentration. In the present study, hemolytic halos were measured over different periods of time, (1, 2, 3, and 14 h). The results obtained showed that at 14 h halos generated measured 19.2 ± 1.5 and 17.00 ± 1.2 mm for *C. molossus molossus* and *C. m. molossus nigrescens*, respectively, whereas at the same concentration over a longer period of time (24 h) with the venoms of *C. triseriatus* and *C. ravus* generated smaller halos 15.00 ± 0.0 (CT1), 12.33 ± 0.58 (CT2), 16.67 ± 0.58 (CR3), and 0.00 ± 0.00 (CR4), showing the hemolytic potential of these species is lower.

On the other hand, Pirela et al., in 2006, determined that the indirect hemolytic dose of *Crotalus durissus cumanensis* venom to produce a 20 mm hemolytic halo was 379.51 ± 67.67 µg of venom [16]. In a similar study, Dos Santos et al. in 1993 obtained a dose of approximately 310 µg for the white venom and 350 µg for the yellow venom of *Crotalus durissus ruruima* to produce hemolytic halos of 20 mm [26]. With respect to *Crotalus triseriatus* and *Crotalus ravus* venoms, an average of 18.67 ± 1.53 mm was obtained at 100 µg/mL concentration of venom. Although there are no equivalent values in the measurements of hemolytic halos, the venom of *C.triseriatus* and *C. ravus* have close values in the measure of their halos in comparison with the other studies and in a lower venom concentration.

In accordance with Macías-Rodríguez et al., in 2014 [27], during the fall months, there exists a high proteomic concentration in rattlesnake venom. *C. ravus* and *C. triseriatus* were sampled in September and November, respectively, months which correspond to the autumn, while the individuals sampled by Pirela et al. in 2006 [16] were sampled in May, June, and July, months that have been shown to have decreased protein concentration. On the other hand, in 2010 Chippaux et al., [6] reported that the species *C. durissus durissus* and *C. durissus terrificus* have myotoxic and neurotoxic venoms compared to other species of the genus *Crotalus*, which mostly have hemotoxic and histologic venoms [28]. Therefore, due to this, in *C. triseriatus* and *C. ravus*, the highest concentration evaluated in this study (100 µg/mL) was enough to produce halos with measurements similar to those of the aforementioned study.

Treatment CR4, characterized by its transparent color, did not show hemolytic activity. This variation in color has been observed in other viperids. In the study carried out by Macías-Rodríguez et al., in 2014 [25], *C. molossus* presented a yellowish venom which turned out to be more hemolytic than *Crotalus tigris* venom, which was transparent in appearance, similar to *C. ravus* (CR4). Galán et al., in 2004 [29], reported that yellowish venoms have greater toxicity compared to white venoms. Lourenço et al., in 2013 [30], reported that the yellow coloration of the venom is due to the presence of crotamine, a myotoxin from rattlesnakes.

Snake venom complexity produces a source of bioactive molecules with different activities. The results obtained in this study confirm rattlesnake’s crude venom contains compounds that could be used as therapeutic models, in this case, molecules with antibacterial activity. Although the venom cannot be used directly due to its high toxicity, some of its compounds will serve as prototypes for the development of new drugs. 

## 4. Conclusions

Until today, there are no studies reporting on the antibacterial and hemolytic activity of the venoms of *C. triseriatus* and *C. ravus*. The results of the present study indicate that both rattlesnakes produce venoms rich in bioactive compounds with a bactericidal effect against *Pseudomonas aeruginosa*. These compounds could also serve as new antimicrobial drugs for the treatment of diseases caused by this bacterium; however, the isolation, identification, and evaluation of these molecules is necessary since it could present hemolytic activity. 

## Figures and Tables

**Table 1:** Individual data of trapped rattlesnakes in Hidalgo State.

Species	Species Characteristics	Individual Identification	Gender	Age	Length (cm)	Weight (g)
*Crotalus triseriatus*	Triangular head 8–10 rattles Postocular strip	CT1	Male	Adult	37	210
		CT2	Male	Adult	25	175
*Crotalus ravus*	Triangular head Thin rattle Symmetric scales in head	CR3	Male	Adult	25	180
		CR4	Male	Adult	25	175

**Table 2:** Minimal Inhibitory Concentration (MIC) and Minimal Bactericidal Concentration (MBC) of *Crotalus triseriatus and Crotalus ravus* venoms.

Evaluated Bacteria	Evaluated Treatments µg/mL (MIC/MBC)				Controls (MIC/MBC)	
	CT1	CT2	CR3	CR4	Nutritive Broth	Kanamycin (µg/mL)
*E. coli*	-	-	-	-	-	2/4
*P. aeruginosa*	-	50 ^a^/100 ^A^	50 ^a^/100 ^A^	-	-	16 ^b^/64 ^B^
*S. aureus*	-	-	-	-	-	1/4

CT1 *Crotalus triseriatus* 1, CT2 *Crotalus triseriatus* 2, CR3 *Crotalus ravus* 3*,* CR4 *Crotalus ravus* 4 ^a,b^ Different small letters indicate significant statistical differences between MIC (*p* < 0.05) ^A,B^ Different capital letters indicate significant statistical differences between MBC (*p* < 0.05)

**Table 3:** Hemolysis halos generated by *C. triseriatus* and *C. ravus* venoms.

Concentration (µg/mL)	Evaluated Treatments					
	CT1	CT2	CR3	CR4	Nutritive Broth	Tween 80
100	18.67 ± 1.53 ^a,A,^*	17.00 ± 1.00 ^a,A^	18.67 ± 1.15 ^a,A,^*	0.0 ^b^	0.00	20.33 ± 0.58 *
50	15.00 ± 0.0 ^b,B^	12.33 ± 0.58 ^c,B^	16.67 ± 0.58 ^a,B^	0.0 ^d^		
25	13.67 ± 0.58 ^a,B,C^	13.67 ± 1.15 ^a,B^	13.33 ± 0.58 ^a,C^	0.0 ^b^		
12.5	12.00 ± 1.00 ^a,C^	10.00 ± 0.00 ^a,b,C^	10.33 ± 0.58 ^b,D^	0.0 ^c^		
6.25	8.67 ± 0.58 ^a,D^	8.33 ± 0.58 ^a,C^	7.33 ± 0.58 ^a,E^	0.0 ^b^		
3.12	0.00 ± 0.00 ^a,E^	0.00 ±0.00 ^a,D^	0.00 ± 0.00 ^a,F^	0.0 ^a^		

^a,b,c^ Different letters indicate significant statistical differences between treatments (*p* < 0.05). ^A,B,C^ Different letters indicate significant statistical differences between concentrations (*p* < 0.05). * No statistical differences between treatments (*p* > 0.05). CT1 *Crotalus triseriatus* 1, CT2 *Crotalus triseriatus* 2*,* CR3 *Crotalus ravus* 3, and CR4 *Crotalus ravus* 4.
